# Abiotrophia Causing Prosthetic Joint Septic Arthritis

**DOI:** 10.7759/cureus.22801

**Published:** 2022-03-03

**Authors:** Juliana N Young, John York

**Affiliations:** 1 Department of Internal Medicine, Burrell College of Osteopathic Medicine, Las Cruces, USA; 2 Department of Orthopedic Surgery, Rehoboth McKinley Christian Health Care Services, Gallup, USA

**Keywords:** enterobacter cloacae, 16s rdna pcr, prosthetic joint infection, abiotrophia defectiva, charcot marie tooth disease

## Abstract

A 71-year-old Caucasian male with a past medical history of Charcot-Marie-Tooth disease type 2 presented to our rural hospital for left knee pain, swelling, and difficulty walking. The patient had prior bilateral total knee replacements with a subsequent left knee revision due to infection. Joint aspiration was culture-positive and 16S recombinant DNA (rDNA) sequence positive for *Abiotrophia defectiva*. The patient underwent a left total knee extraction with a temporary antibiotic spacer insertion. On discharge, the patient received an initial six weeks of ceftriaxone 2g IV. At the outpatient six-week follow-up, the patient was cleared of the *Abiotrophia *infection. However, later complications and a subsequent *Enterobacter cloacae* infection arose.

## Introduction

The risk of a general prosthetic joint infection (PJI) is 2% [1] and is typically caused by biofilm-forming organisms such as *Staphylococcus *and *Streptococcus*. However, septic arthritis can also be caused by atypical organisms. In such cases, the identification of the atypical organism is of paramount importance to determine the most effective therapeutic approach. *Abiotrophia defectiva* is a rare cause of bacterial endocarditis. It remains difficult to identify by culture, taking five days to grow [2]. Mass spectrometry and 16S recombinant DNA (rDNA) polymerase chain reaction (PCR) have been previously used to identify *Abiotrophia *infections [3]. However, identification, and thus treatment, can be further delayed in resource-limited hospitals by a lack of diagnostic equipment and personnel. 

To our knowledge, only six cases of prosthetic joint infection due to *Abiotrophia *have been reported as of 2020 [3-5]. Here we report an additional case of *Abiotrophia *infection of a prosthetic joint in a patient with Charcot-Marie-Tooth (CMT) disease. 

## Case presentation

A 71-year-old Caucasian male presented to our rural emergency department for left knee pain, swelling, and difficulty walking that started earlier that day. He had received bilateral total knee replacements five years ago and had a previous left knee revision due to an infection with an unknown etiology three years prior. The patient had no known trauma, but he had a recent left foot cellulitis, which was successfully treated with Augmentin a few weeks prior to presentation. The patient had a colonoscopy in the past year with antibiotic prophylaxis and had dental work performed without antibiotic prophylaxis three months prior. 

**Figure 1:**
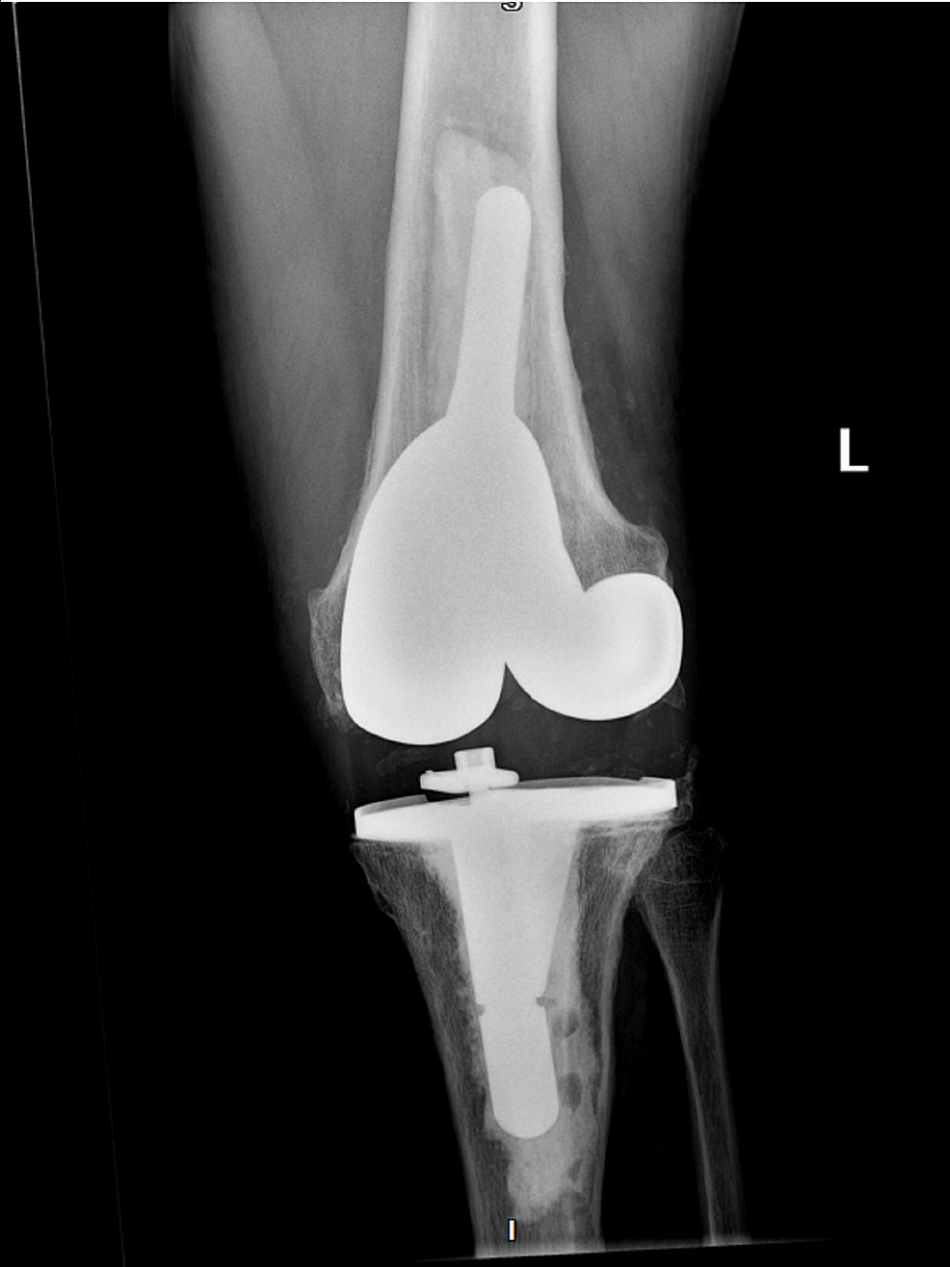
X-ray of the infected left knee with total arthroplasty prior to explantation (anteroposterior)

**Figure 2:**
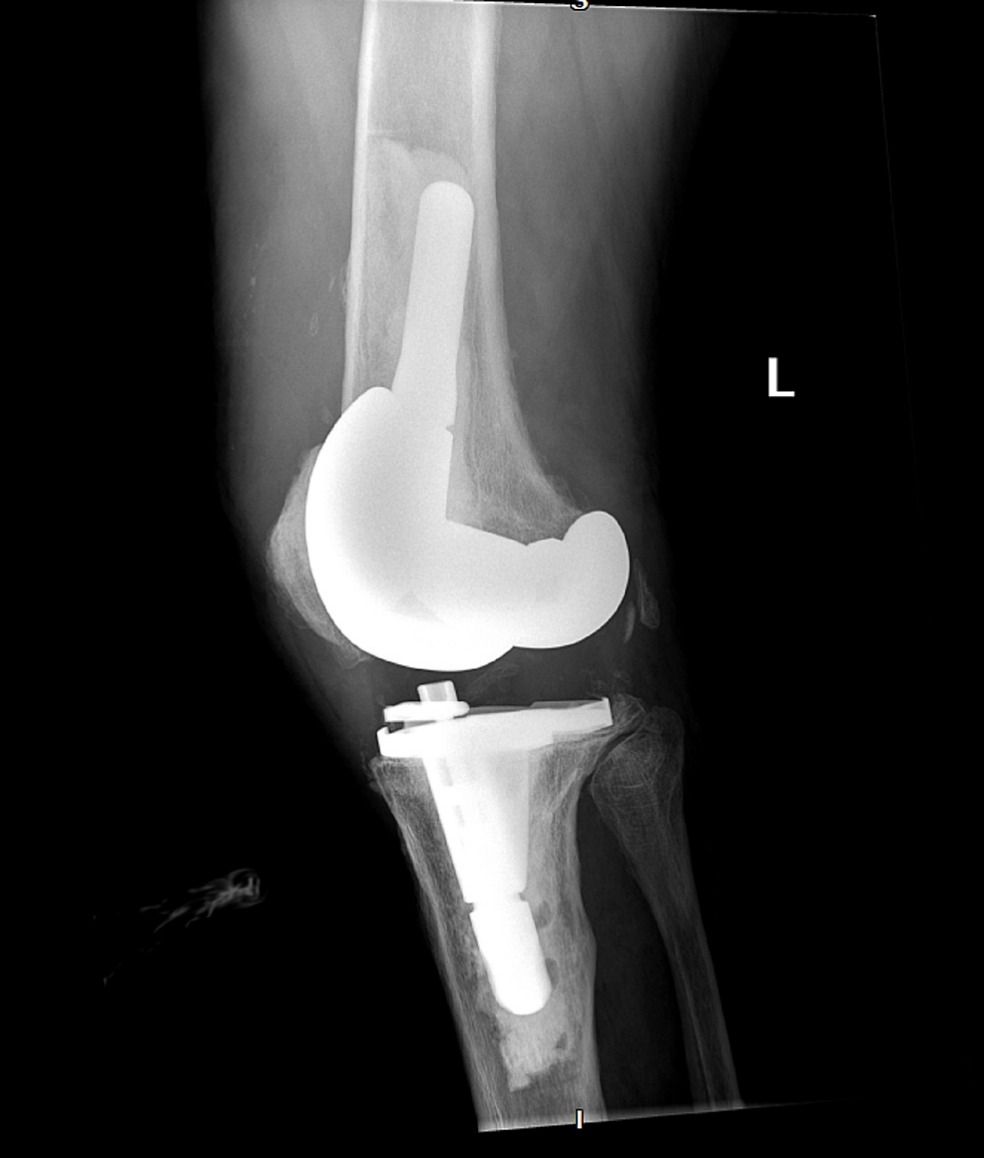
X-ray of the infected left knee with total arthroplasty prior to explantation (lateral)

**Figure 3:**
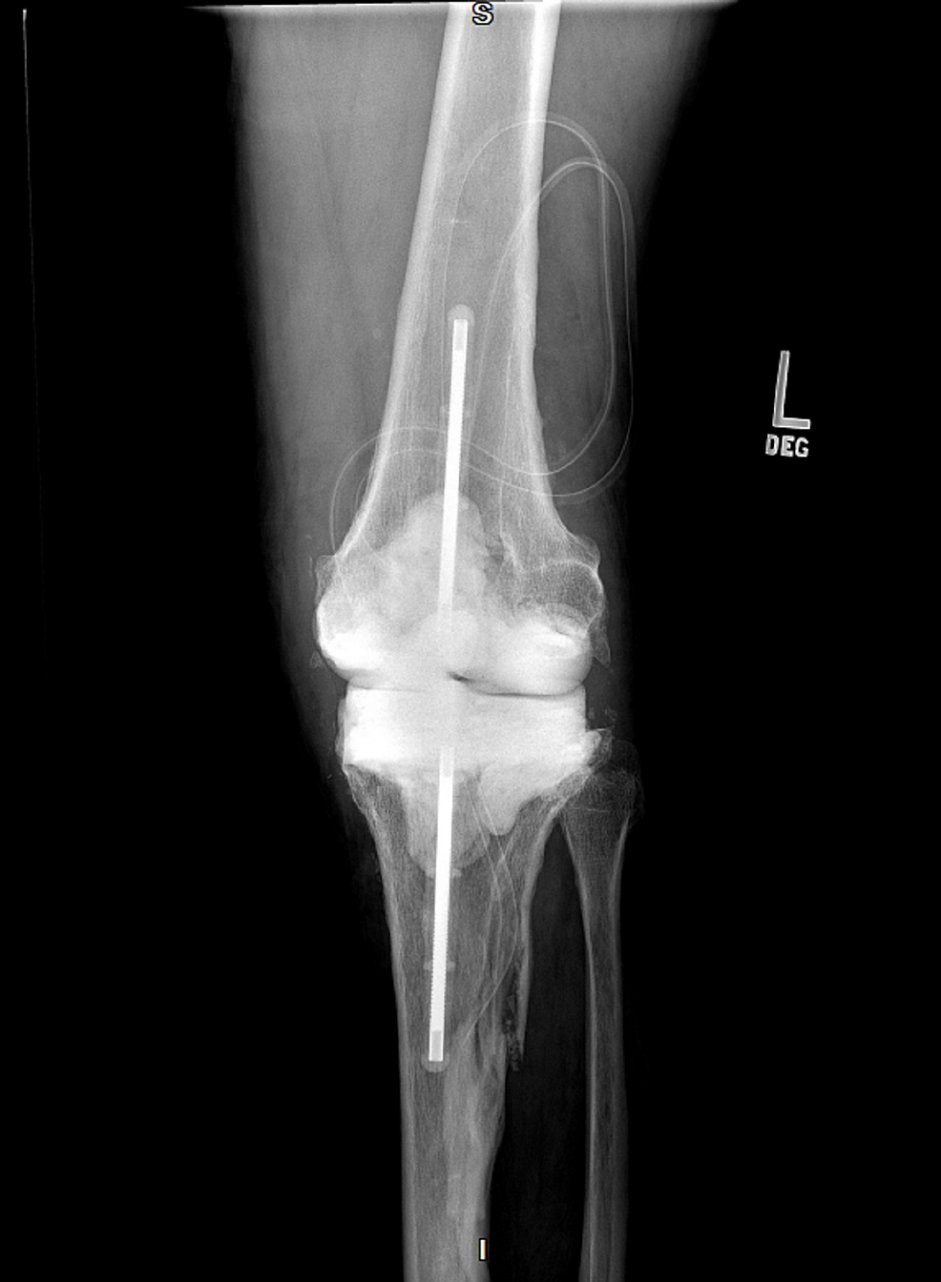
X-ray of the infected left knee post-surgical antibiotic knee spacer (anteroposterior)

The patient has a past medical history of Charcot-Marie-Tooth disease type 2 diagnosed in his thirties. He had retained sensation in his feet but had bilateral Charcot arthropathy and impaired proprioception. The patient had concurrently developed restless leg syndrome. Upon physical examination, the patient was afebrile and normotensive with a normal sinus rhythm and respiratory rate. His left knee was radiating heat and had moderate tenderness and swelling with diminished flexion range of motion. There was a medium-sized left knee joint effusion. Figures 1-2 show initial x-rays of the infected left knee with total arthroplasty. The patient was neurovascularly intact distally. The patient’s C-reactive protein (CRP) was elevated at 14.7 mg/L (normal range: <10mg/L). The joint was aspirated in the ED and revealed brown, cloudy fluid with WBC 5,6917/μL and preliminary gram-negative anaerobic bacilli. He was thus started on one dose of ceftriaxone 2g IV and vancomycin 1500mg IV twice a day (BID). On the second day of admission, the orthopedic surgery department performed a Bactrim washout of the knee with arthroscopic incision and drainage followed by tissue culture. The cultures from the arthroscopic incision and drainage showed gram-negative and gram-positive bacilli with no definitive organism. The patient was then switched from ceftriaxone to a seven-day course of cefepime 2g IV three times a day (TID) for broader gram-negative coverage. The patient was continued on the same dose of intravenous vancomycin for a total of five days prior to transitioning to linezolid 600mg BID for four days for broader gram-positive coverage. Due to the limited resources of our rural facility and inability to further identify the infectious agent, samples were sent out for 16S rDNA sequencing, resulting in delayed organism identification. Our Infectious Disease Department, therefore, recommended an explanation of the patient’s total knee replacement with implantation of a temporary antibiotic vancomycin/gentamicin spacer (Figure 3). PCR sequencing later revealed the causative organism to be the gram-variable coccobacilli *Abiotrophia defectiva*, explaining the initial culture results of the gram-positive and gram-negative bacilli. *Abiotrophia defectiva* sensitivities could not be performed as the bacteria was not viable for susceptibility testing. The patient was discharged on six weeks of ceftriaxone 2g IV administered via Groshong catheter. Two weeks following completion of the IV antibiotics, synovial fluid, and blood cultures were taken and showed that the patient was free of the *Abiotrophia *infection.

**Figure 4:**
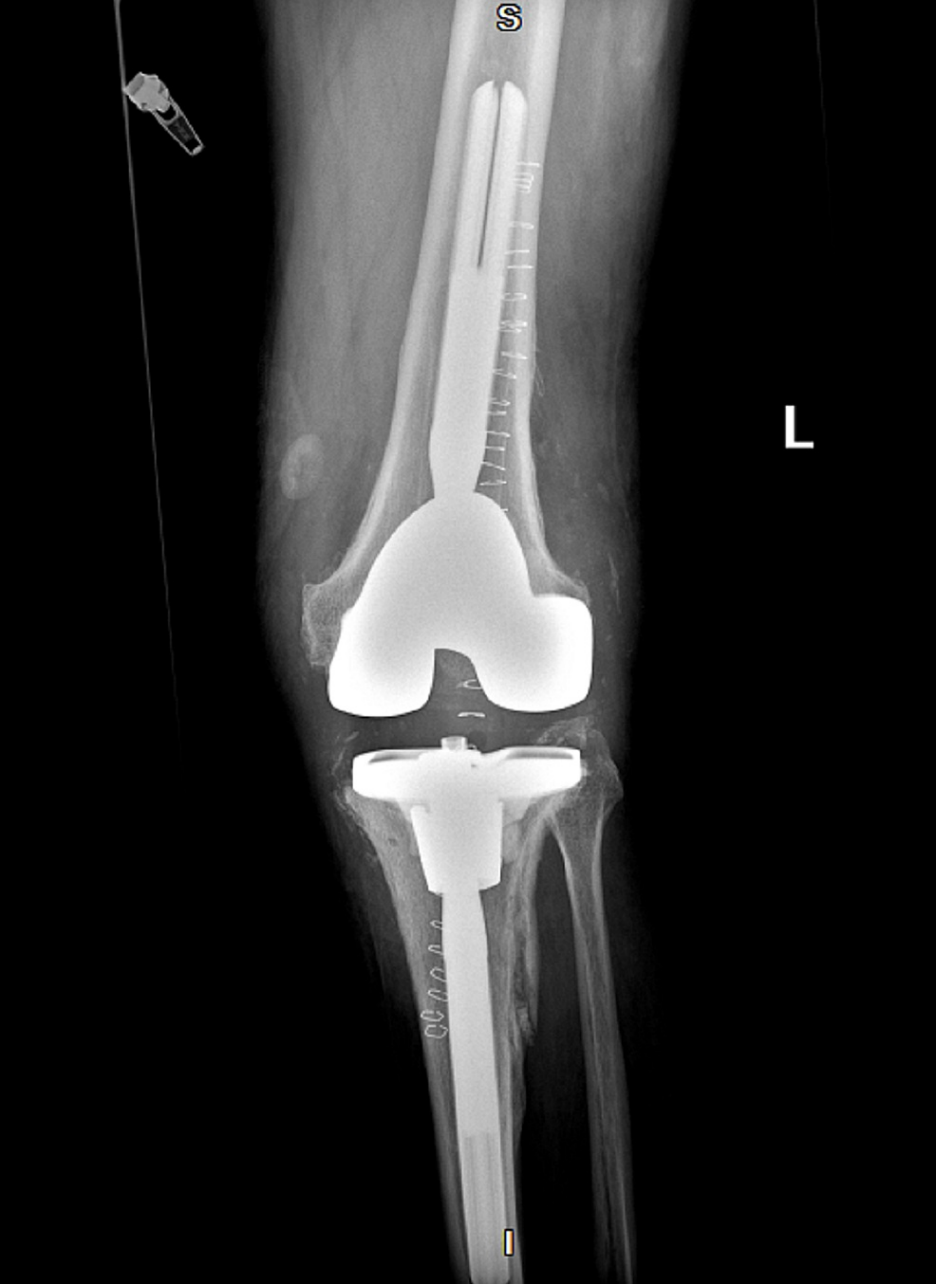
X-ray of the left knee post-antibiotic spacer removal and reimplantation of permanent total knee replacement following the resolution of Abiotrophia infection (anteroposterior)

**Figure 5:**
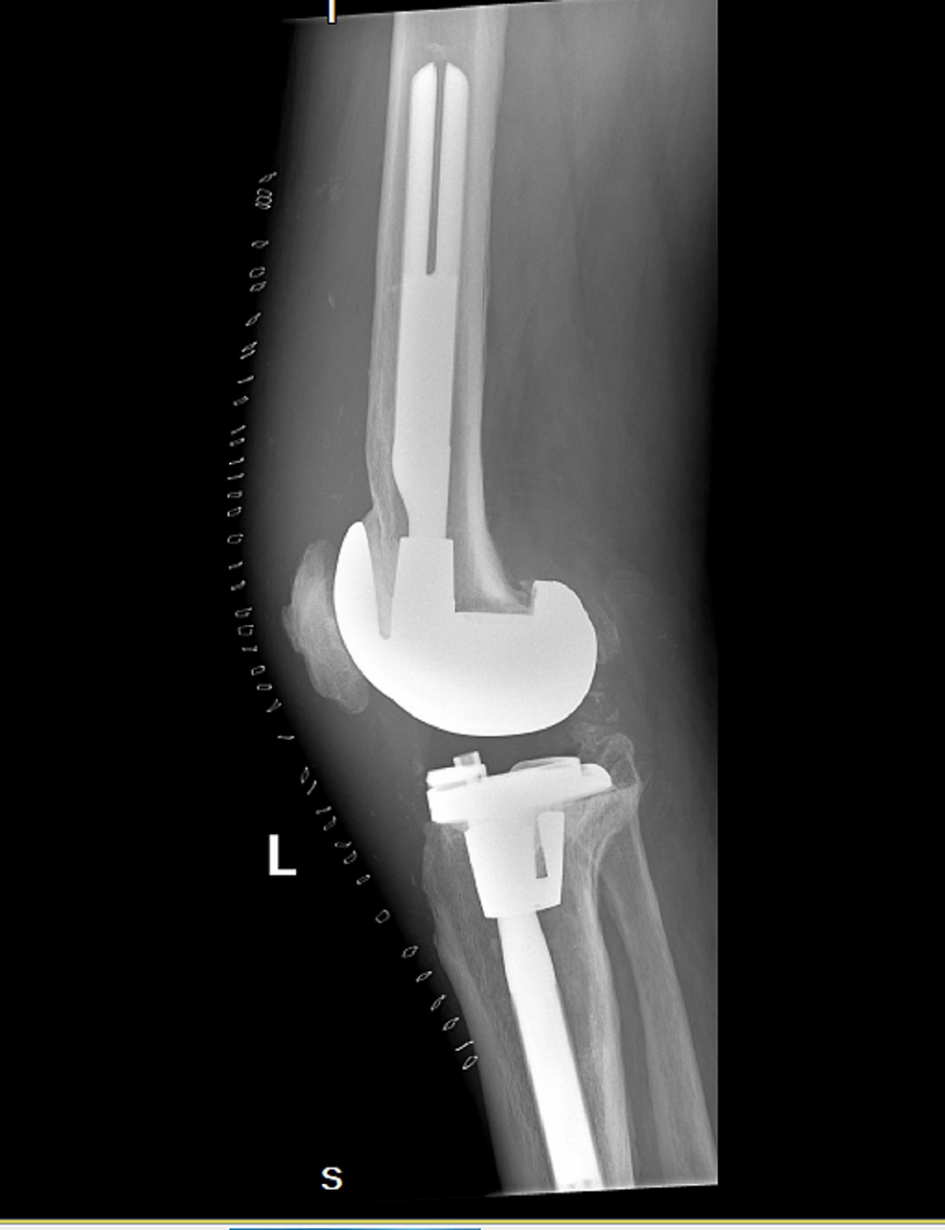
X-ray of the left knee post-antibiotic spacer removal and reimplantation of permanent total knee replacement following the resolution of Abiotrophia infection (lateral)

After the absence of the *Abiotrophia *infection was confirmed, the patient underwent antibiotic spacer removal with a left knee revision replacement (Figures 4-5). The patient was discharged on prophylactic oral amoxicillin 1g TID for six months. However, the amoxicillin was discontinued after several days due to diarrhea. Fourteen days post left knee revision, the patient developed signs of recurrent septic arthritis and returned to our ER. Synovial fluid aspirate was positive for *Enterobacter cloacae* infection with sensitivities to cefepime, meropenem, ciprofloxacin, levofloxacin, and trimethoprim/sulfamethoxazole. The patient was transferred to an unaffiliated urban facility for multiple Bactrim washouts, but tissue samples were negative for growth. The patient was discharged from the unaffiliated facility on an unknown dose of cefepime and metronidazole. Three weeks later, the patient was seen again at the emergency department of the unaffiliated, urban facility for erythema and swelling of the left knee. However, aspiration of the joint showed a bloody synovial fluid with no growth and was diagnosed as bursitis on top of possible cellulitis. The patient was later evaluated to also have a left patellar tendon tear. Since there was a possible residual infection, he was switched to ertapenem 1g IV daily for six weeks and then started on oral levofloxacin 750mg daily. The ertapenem was implemented due to the possibility of developing an extended spectrum beta-lactamase-resistant bacteria with cefepime antibiotics. The patient will be on a lifetime dose of levofloxacin to prevent recurrence of infection. Our orthopedic department will continue to monitor for signs and symptoms of septic arthritis and order a monthly basic metabolic panel (BMP), complete blood count (CBC), erythrocyte sedimentation rate (ESR), and C-reactive protein (CRP) levels. The patient is currently in physical therapy and now awaiting repair of a torn left patellar tendon.

## Discussion

*Abiotrophia defectiva* is a type of nutritionally variant streptococci (NVS) that is part of the normal human microbiome. It is found in the oropharynx and the gastrointestinal and urogenital tracts. It is a significant cause of bacteremia and infective endocarditis with high morbidity and mortality [6]. As previously detailed [3-5], there have only been a handful of reported cases of *Abiotrophia defectiva* causing joint infections. Our literature review revealed a total of nine cases of *Abiotrophia defectiva* knee infections, seven of which occurred in prosthetic knees. 

The presentation of our patient's septic arthritis appears to be idiopathic, but multiple potential causes of infection can be considered. Charcot-Marie-Tooth disease is an inherited neuromuscular disorder with a progressive course [7]. Although it is not immunocompromising, muscular dystrophies and neuropathies may contribute to increased foot injury, as seen with the patient's recent history of foot cellulitis, and may have provided a mechanism for bacterial seeding. The bilateral total knee replacements seen in this patient were also most likely hastened by the increased stress placed on the knees due to CMT-induced muscle weakness causing knee hyperextension. As seen with previously reported cases, the majority of known *Abiotrophia defectiva* joint infections have occurred in total knee replacements [4] since medical prosthetics increase the risk of future infection.

The patient also had a colonoscopy and dental work performed in the past several months. The patient was given antibiotic prophylaxis for his colonoscopy per surgeon preference but did not receive prophylaxis for his dental work. The current guidelines regarding antibiotic prophylaxis for dental work are controversial [1]. Prior to 2015, antibiotics were given to all patients with total hip or knee replacements within two years of implantation. Currently, the American Dental Association advises against prophylaxis, while the American Academy of Orthopedic Surgeons recommends amoxicillin oral prophylaxis in certain scenarios for high-risk patients as defined in the 2016 "Management of Patients with Orthopaedic Implants Undergoing Dental Procedures" [1,8]. According to the American Academy of Orthopaedic Surgeons, patients are considered high-risk when they are immunocompromised, have uncontrolled diabetes (HbA1c >8, or glucose=200 ), or had a previous prosthetic joint infection requiring surgery [8]. As *Abiotrophia defectiva* is known to reside in both the gastrointestinal tract and the oropharynx, it is possible either of these procedures could have introduced the bacteria.

It should be noted that there were multiple demographic and social factors that influenced this case. The rural nature of our hospital required the initial synovial fluid samples to be sent to another lab which contributed to a delay in diagnosis and appropriate treatment. PCR and mass spectrometry are faster and more accurate methods for the diagnosis of *Abiotrophia defectiva *[3] but were unavailable to the laboratory at our hospital.

The impact of the multiple knee surgeries on the patient's quality of life should also be noted. The use of temporary antibiotic spacers required the patient to place minimal body weight on his knees, limiting his ambulation. Due to our patient's age and history of CMT, future surgeries involving the removal of the spacers and implantation of a new total knee increased the risk of further complications such as the development of the *Enterobacter cloacae* infection and left patellar tendon tear.

## Conclusions

Only a small number of cases of *Abiotrophia defectiva* causing prosthetic joint infection have been reported. The current case is unique because of the CMT co-morbidity that may have contributed to the *Abiotrophia defectiva* joint infection in this patient. The historical difficulty of identifying *Abiotrophia defectiva* via culture has been minimized in the past decade by novel technologies, such as 16S rDNA PCR. However, such technologies are not readily available in all hospitals, especially in rural settings, which can delay effective treatment. This case report emphasizes the importance of a thorough look through patient history to determine potential sources of infection, including the oropharynx and gastrointestinal or genitourinary tract. Atypical joint infections should be considered in the differential diagnosis of patients with prosthetic joints and significant co-morbidities, as in this case.
